# Extended endoscopic endonasal approach for resecting anterior intrinsic third ventricular craniopharyngioma

**DOI:** 10.3389/fonc.2022.998683

**Published:** 2022-09-29

**Authors:** Yuefei Zhou, Jialiang Wei, Tao Jin, Yue Hei, Pengfei Jia, Jincai Lin, Shuangwu Yang, Xiaofan Jiang, Weiping Liu, Dakuan Gao

**Affiliations:** ^1^ Department of Neurosurgery, Xijing Hospital, Fourth Military Medical University, Xi’an, China; ^2^ Department of Health Service, Fourth Military Medical University, Xi’an, China; ^3^ Department of Neurosurgery, An Kang Center Hospital, An Kang, China; ^4^ Department of Neurosurgery, Mao Ming People’s Hospital, Mao Ming, China

**Keywords:** extended endoscopic endonasal approach, intrinsic third ventricular craniopharyngioma, translamina terminalis, skull base reconstruction, *in situ* bone flap

## Abstract

**Background:**

The surgical treatment of the extended endoscopic endonasal approach (EEEA) is a safe and effective treatment for suprasellar craniopharyngiomas. However, due to damage to the hypothalamus and third ventricle floor (TVF), EEEA is generally regarded as unsuitable in treating intrinsic third ventricle craniopharyngioma (ITVC) that is entirely within the third ventricle. Until now, there have been only a small number of reports using EEEA to treat TVC *via* a supra-infrachiasmatic approach. Given that the translamina terminalis (TLT) corridor was used in the transcranial subfrontal approach, EEEA *via* a suprachiasmatic approach may be feasible and practical to treat ITVC. In the current study, we accumulated experience applying the suprachiasmatic translamina terminalis (STLT) corridor for anterior treatment of ITVC.

**Methods:**

From March 2016 to December 2020, 14 patients with ITVC in our center were analyzed retrospectively. All patients underwent surgery by EEEA *via* an STLT corridor. The multilayer reconstruction technique was adopted to achieve skull base reconstruction. Data concerning the patient’s tumor resection, vision, hypophyseal hormone, and complications were collected.

**Results:**

Gross-total resection was achieved in 13 (92.8%) of14 patients, with achievement of near-total (90%) resection in the remaining 1 patient. Nine cases (64.3%) were papillary craniopharyngiomas, and the other 5 cases were adamantinomatous subtypes. Postoperatively, 3 patients with pituitary insufficiency received hormone replacement therapy. No permanent diabetes insipidus or hypothalamic obesity was found. All pairs showed significant improvement or stability in vision except 1 patient who encountered visual deterioration. No other neurological deficit occurred postoperatively. Observation results for the exudation of nasal tissue and the length of hospitalization were satisfactory. After a mean follow-up period of 26.2 months, tumor recurrence was not observed.

**Conclusion:**

TLT is a minimally invasive corridor used in EEEA for treating anterior ITVC without increasing risks of visual and hormonal deficits. The multilayered reconstruction technique we used is a safe and effective method for achieving watertight closure and avoiding cerebrospinal fluid leaks and infection. The endonasal approach *via* STLT provides a new, safe and efficacious operative strategy that should be considered a surgical alternative in treating ITVC.

## Introduction

Craniopharyngioma is a common type of nonmalignant epithelial tumor that develops in the suprasellar area and originates along the skull base midline. Previously, the transcallosal approach, transcortical transventricular approach, pterional approach with incision of the lamina terminalis, and any combination of these approaches have been proven to be effective in the removal of intrinsic third ventricle craniopharyngioma (ITVC) (1–3). The evolution of extended endoscopic endonasal approach (EEEA) has resulted from medical technology development, improved anatomical understanding and growing collaboration between otolaryngology and neurosurgery. EEEA uses a natural corridor to fully expose the tumor without brain tissue retraction, which causes less injury and allows faster recovery (4). Thus, EEEA is a safe and effective alternative for the treatment of certain craniopharyngiomas in locations ranging from the sellar region to the third ventricle without extralateral extension (5). However, for some craniopharyngiomas, especially for those that anchor deep and adhere to vital structures, such as third ventricle craniopharyngioma (TVC), one challenging subtype that localizes entirely within the third ventricle (purely intraventricular) (6, 7), the standard endoscopic endonasal approach (EEA) is thought to be not perfectly suitable, as it could exert undesirable harm on adjacent structures such as the ventricle floor and hypothalamus (8). Therefore, we explored a more minimally invasive surgical corridor to incise the TVC.

For suprasellar craniopharyngioma that breaks through the third ventricle floor (TVF), complete resection of the tumor is achieved *via* the supra-infrachiasmatic approach (9). Previously, the stretched ventricular floor was sharply incised through the corridor between the anteriorly displaced chiasm and the stalk, allowing visualization of ITVC (10). Sometimes, the technique of resecting the posterior clinoid or pituitary transposition (11) to create more room may help manage TVC with great upper and anterior extension. Nevertheless, incision of the TVF is the main cause of postoperative complications, and the ventricular floor and pituitary stalk are often partially or totally sacrificed when resecting the tumor. Because the subfrontal translamina terminalis (TLT) corridor has been used in transcranial procedures to treat TVC and has shown promising results (12, 13), the suprachiasmatic translamina terminalis (STLT) corridor, which opens the lamina terminalis (LT) for better exposure of the anterior third ventricle and avoids ventricle floor incision, could be a new neuroendoscopic surgery option for neurosurgeons (14). However, few reports have shown the efficacy and prognosis of EEEA in treating ITVC *via* the TLT corridor.

In light of the controversy concerning the operative management of craniopharyngiomas that underwent EEEA, we collected clinical data from anterior ITVC patients. The current manuscript reports our preliminary experience *via* the STLT corridor in a series addressing this rare subset.

## Materials and methods

### Ethics statement

The studies involving human participants were reviewed and approved by Ethics Committee of Fourth Military Medical University. The written informed consent was obtained from participants enrolled in this study.

### Patient population

In this retrospective study, we reviewed patients in a single institution (Department of Neurosurgery, Xijing Hospital, Fourth Military Medical University, Xi’an, China) who underwent EEEA for anterior TVC from March 2016 to December 2021. The information collected from the patients’ electronic medical records included demographics, presenting symptoms, ophthalmological exams, operative notes, postoperative courses, histopathological diagnoses, laboratory data and image files (**Table 1**).

**Table 1:** Pre- and intra-operative clinical characteristics of patients.

*#*	*Age/Sex*	*Pre-op symptoms*	*Endocrine deficit*	*BMI*	*Adherence site/ Type*	*Diameter/Vol (cm/cm3)*
***1***	29, M	VD, HA	Partial API(HG)	24.5(+0.4)	Tuberculum / loose	2.9/10.1
***2***	43, F	VD	None	25.8(+1.2)	Tuberculum/loose	2.2/8.5
***3***	33, F	HA, VD	Partial API (HG)	21.7(-0.5)	Infundibulum/loose	3.2/11.7
***4***	38, M	HA, Sz	None	28.6(+2.7)	Infundibulum /loose	1.9/6.3
***5***	61, M	ML,	None	23.1(-0.2)	Tuberculum /loose	1.1/2.8
***6***	45, M	HP, HA	Partial API (HG, HT)	30.1(+3.9)	Tuberculum /loose	3.4/12.6
***7***	51, F	HA	DI	27.4(+2.6)	Infundibulum /loose	2.8/9.9
***8***	47, F	HA, ML	None	22.8(+0.8)	Infundibulum /loose	2.1/5.6
***9***	49, M	VD	Partial API (HG, HT)	25.5(+1.4)	Infundibulum /tight	3.1/12.4
***10***	52, M	VD	None	29.7(+1.4)	Tuberculum / loose	2.8/11.2
***11***	54. M	HA	Partial API (HG)	21.9(+0.9)	Tuberculum /loose	2.1/7.3
***12***	43, F	HA	None	24.6(+1.8)	Infundibulum/loose	1.6/5.2
***13***	60, F	VD	None	20.6(-0.9)	Tuberculum /loose	2.3/7.6
***14***	44, M	ML	None	24.7(-1.1)	Infundibulum/loose	1.1/3.1

API, anterior pituitary insufficiency; DI, diabetes insipidus; HA, headache; HG, hypogonadism; HP, hyperphagia; HT, hypothyroidism; ML, memory loss; Sz, seizure; VD, visual deterioration.

Postoperative changes are shown in parentheses in the BMI column.

### Imaging analysis

**Figure 1:**
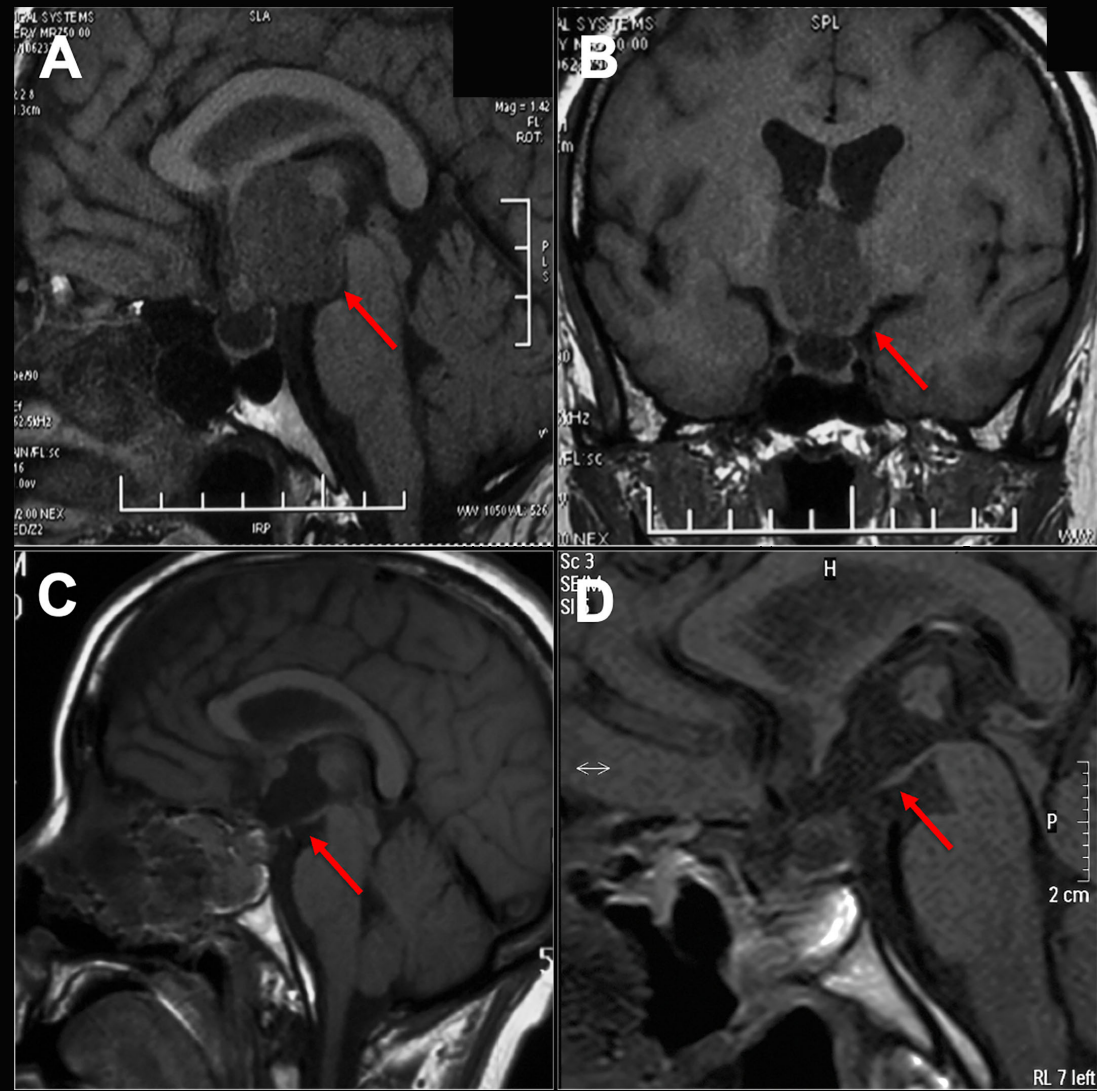
Preoperative and postoperative imaging of craniopharyngioma. **(A, B)** An intact third ventricular floor structure could be seen preoperatively and was considered an intrinsic third ventricular craniopharyngioma. **(C, D)** The tumor was completely incised by EEEA via the suprachiasmatic translamina terminalis corridor, and the intact third ventricular floor was well visualized postoperatively on sagittal images.

The imaging and volumetric assessment and analysis were independently performed by an experienced neuroradiologist with access to all imaging sequences. Computed tomography is useful for demonstrating calcification, the degree of pneumatization and the locations of septations in the sphenoid sinus. MRI scanning was performed before surgery to provide excellent details about the tumor’s size and location and to analyze the topology of the tumor with pituitary, optic chiasma (OC) and TVF. The ITVC was defined as solely occupying the confines of the third ventricle on axial, sagittal, and coronal images. Additionally, the intact and inferiorly displaced papillary body is indirect evidence suggesting that the tumor may be completely located in the third ventricle (**Figures 1A, B**). Based on magnetic resonance imaging (MRI) analysis, we only included anterior ITVC patients, and craniopharyngiomas located supradiaphragmatically protruding into the third ventricle were excluded from the research. Tumor origin and location were further confirmed intraoperatively. The position of the OC, anterior communicating artery (AComA) and the size of lateral ventricles on MRI also enabled us to choose the surgical approach.

### Management strategy

Broad-spectrum antibiotic therapy and hydrocortisone sodium succinate were administered perioperatively for preventing peri-operative infection and pituitary hypofunction. Mannitol was applied for dehydration to reduce intracranial pressure. The patients were strictly placed in a semisitting position to lower the intracranial pressure hence the likelihood of CSF leakage. The steps reaching sellar and suprasellar regions *via* EEEA have been extensively described in the literature. Craniopharyngioma removal *via* EEEA follows the same steps of standard transcranial microsurgery, which include identification of the tumor, internal tumor debulking, extracapsular dissection in the arachnoid-capsular plane, protection of the neurovascular structures (including suprasellar perforating arteries), and intraoperative decision-making pertaining to leaving residual tumor if excessive dissection is likely to result in neurological morbidity. Intratumoral decompression is the most important step, and the capsule margin can be mobilized after enough debulking of the tumor. Meanwhile, cerebrospinal fluid leakage and insufficiency of skull base reconstruction remain the major complications met postoperatively. Therefore, the objectives are to minimize CSF pressure, eliminate graft migration, and promote graft vascularization and granulation tissue formation. The multilayer technique was adopted to achieve skull base reconstruction, and lumbar drainage was not used as a preventive maneuver preoperatively.

Nasal silver gauze was removed 2 weeks after surgery, and nasal endoscopic removal of scabs or scars was performed by ENT doctors 1 month later. MRI was used to observe the degree of tumor resection, and sex hormone and thyroxine hormone tests were used to assess endocrine changes.

### Surgical approach and techniques

The transtuberculum-transplanum-translamina terminalis of EEEA was used for removal of anterior ITVC. Patients were in a supine position with the head rotated to the right side. A fascia lata donor site was also prepared to harvest autologous fascia for cranial base reconstruction if the mucosa was defective. The operation proceeded with a binostril technique. Briefly, one surgeon worked bimanually, while the other drove the endoscope to facilitate 3D perception of the surgical field. The middle turbinate was resected to enhance visibility, and a needle electrode was used to make a pedicled nasoseptal flap, which included the nasal septum and the lateral wall of the nasal cavity. `Adequate access was the first step toward complete resection with preservation of the involved neurovascular structures. A wide sphenoidotomy is important because it provides extra space for passage and instrument manipulation within the deep operative field above the chiasm and minimizes instrument collision. The posterior ethmoid sinus was removed using a Kerrison rongeur to sufficiently expose the planum sphenoidale and tuberculum sellae. It is also important to ensure meticulous hemostasis in the anterior portion of the nasal operation, as it may be a constant source of blood rundown into the surgical field.

Bony septations and sinus mucosa were removed to identify skull base landmarks that are more distinctive, which increases the possibility of flap adherence to the bone in the skull base reconstruction phase. The anatomic landmarks include the planum sphenoidale, tuberculum sellae, bilateral optic canal, opticocarotid recess and sellar floor. The prominence of the landmarks depends on the degree of pneumatization of the sinus. Navigation can be used to compensate the underpneumatized sinus to ensure safety.

The *in situ* bone flap was removed integrally with a high-speed drill, which consisted of planum sphenoidale, tuberculum sellae, partial optic canal and sellar floor. The bone over the carotid arteries and optic canal was drilled in a unique manner by which the bone was drilled with an eggshell-ish appearance so that the bone flap could be obtained more easily with less possibility of damaging the structure beneath. Additionally, copious irrigation was applied to avoid thermal injury to the underlying neurovascular tissues. Rongeur was used, if necessary, to further extend the bone window, and doppler and neuronavigator were used to ensure procedure safety. Venous bleeding is often encountered in this area when removing the bone, but even vigorous venous bleeding can be easily controlled with Surgiflo (Ethicon, America) and gentle pressure.

The dura was incised along the central axis, and the margin of the incision should be smaller than that of the bone defect, especially in the vertical direction, to ensure that the bone flap will not slip into the subdural space during reconstruction. The horizontal diameter can be flexibly adjusted to meet the needs of surgical procedures. A precise opening was required to preserve the dura to support the brain, to minimize the defect and to satisfy the need for reconstruction. The dural edges were cauterized and shrunk to increase visualization. It is also possible to trim dural edges using a Kerrison rongeur when expanding the operative corridor.

**Figure 2:**
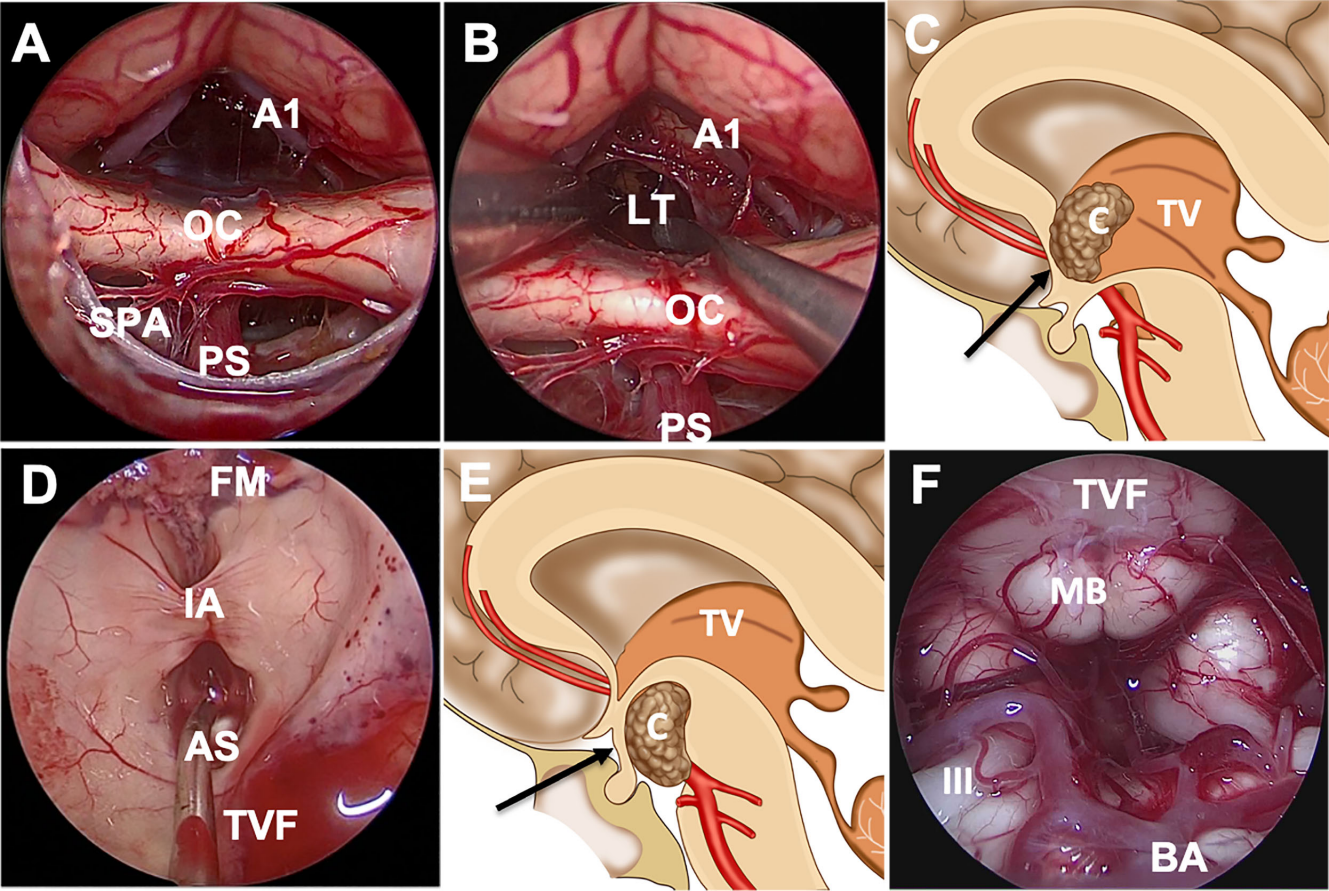
Endoscopic endonasal approach through the suprachiasmatic or infrachiasmatic approach for craniopharyngioma. **(A)** The infrachiasmatic space was narrow, and there was an intact pituitary stalk and third ventricular floor. **(B, C)** Incision of the bulged and elongated lamina terminalis to access the third ventricle through suprachiasmatic corridor. **(D)** The structures in the third ventricle were observed after complete tumor resection. **(E, F)** Schematic diagram of suprasellar craniopharyngioma resection through the infrachiasmatic corridor; the structures were observed after tumor resection. Abbreviations: A1, A1 segment of anterior cerebral artery; AS, aqueduct of sylvius; BA, basilar artery; C, craniopharyngioma; FM, foramen of Monro; IA, interthalamic adhesion; III, oculomotor nerve; LT, lamina terminalis; MB, mamillary body; OC, optic chiasm; PS, pituitary stalk; SPA, superior pituitary artery; TV, third ventricle; TVF, third ventricular floor.

**Figure 3:**
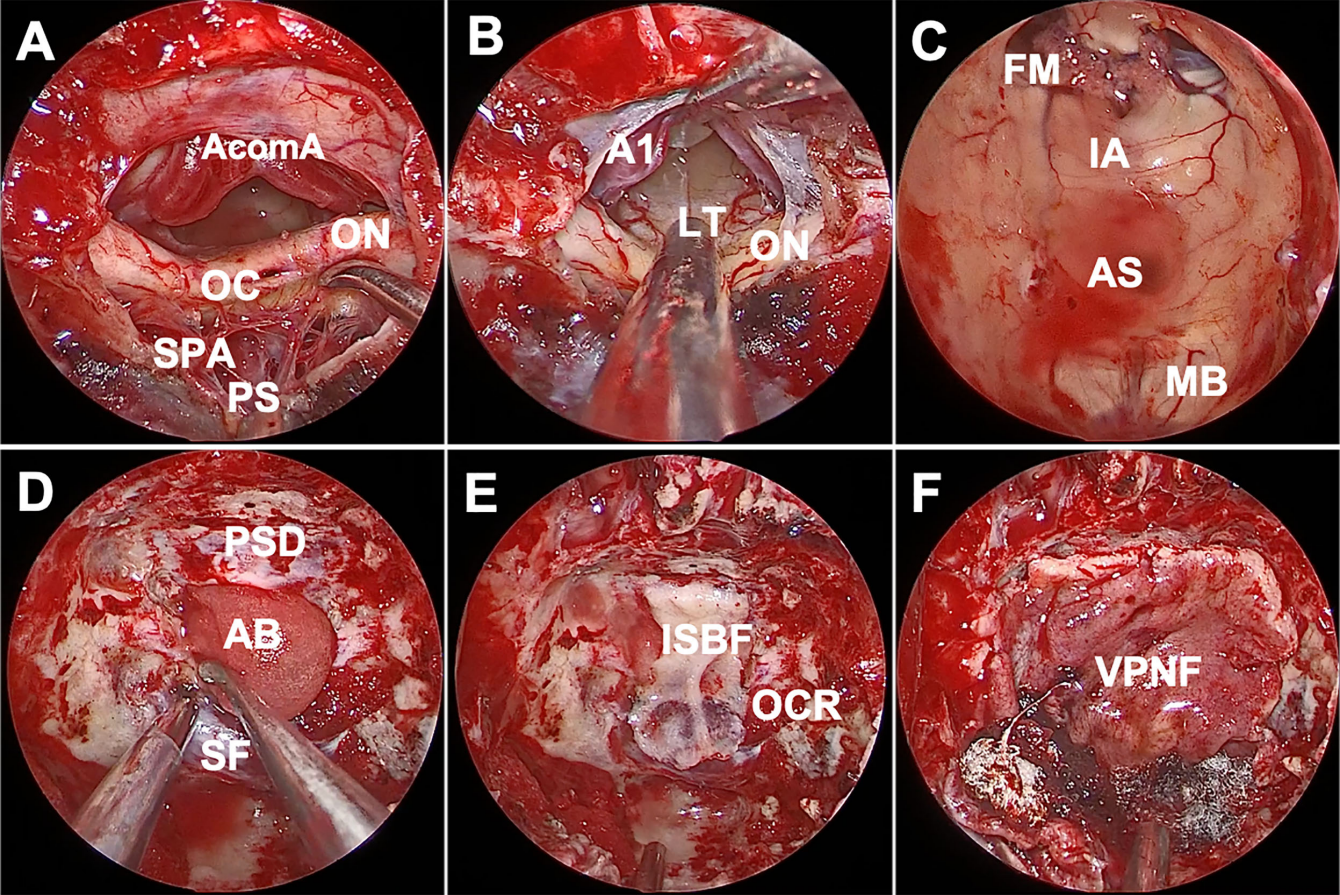
Endoscopic endonasal view of the procedure and nuances of craniopharyngioma surgery. **(A)** The infrachiasmatic corridor was narrow, there were many branches of the superior pituitary artery, intact pituitary stalk and no tumor was detected. **(B)** There was essentially no vascular perforation at the lamina terminalis after opening the suprachiasmatic arachnoid, and we entered the third ventricle via lamina terminalis ostomy. **(C)** The structure of the third ventricle was observed after complete tumor resection, including intact mammillary body. **(D)** An absorbable artificial biomembrane was placed as the first step. **(E)** In situ bone flap was used on the biomembrane to achieve complete osseous reconstruction. **(F)** A vascularized pedicled nasoseptal flap completely repaired the defect. AcomA, anterior communicating artery; OC, optic chiasm; ON, optic nerve; SPA, superior pituitary artery; PS, pituitary stalk; A1, A1 segment of anterior cerebral artery; LT, lamina terminalis; FM, foramen of Monro; IA, interthalamic adhesion; AS, aqueduct of sylvius; MB, mamillary body; PSD, planum sphenoidale dura; AB, artificial biomembrane; SF, sellar floor; ISB, in situ bone flap; OCR, opticocarotid recess; VPNF, vascularized pedicled nasoseptal flap.

After opening the chiasm cistern arachnoid, the locations of the pituitary stalk and OC were identified first. We confirmed intraoperatively that the tumor was completely located in the third ventricle and that no tumor was found in the narrow infrachiasmatic space (**Figure 2A**). Next, the arachnoid was sharply dissected and removed to further open the suprachiasmatic space, and LT ostomy was conducted after uplifting the frontal lobe and AComA complex to expose the tumor (**Figure 2B**). Focusing on the tumor-hypothalamus interface is of fundamental importance to the surgeon. After debulking and removing the tumor using a suction tube, tumor-grasping forceps, endoscopic scissors and ultrasonic surgical aspirator, the residual tumor was separated carefully from the ventricular wall. Most of the papillary subtypes, in our cases, were not closely adherent to the ventricular wall, and some were even free from the ventricle. Overall, directly visualized sharp dissection is paramount to preserve hypothalamic function during surgery. Certainly, subtotal tumor removal is advisable if further dissection is likely to result in unacceptable neurological morbidity. After tumor resection, the surgical areas were repeatedly irrigated with warm water to reduce potential inflammation and to achieve hemostasis. Intraventricular structures, including the foramen of Monro, choroid plexus, papillary body and aqueduct of sylvius, were present in our surgical procedures (**Figure 3C**).

Reconstruction of the skull base is an important procedure. A watertight multilayer skull base reconstruction technique was performed after tumor removal. First, an absorbable artificial biomembrane was placed into the subdural space as the first defense against CSF leaks (**Figure 3D**), and an *in situ* bone flap was used afterward to achieve complete osseous reconstruction (**Figure 3E**). Furthermore, the pedicled nasoseptal flap covered the bone flap for extra reinforcement (**Figure 3F**). Surgicel and gelatin sponges were stuffed around to further support the flap against displacement or migration and to accelerate healing. Silver ion gauze was also used to fill the nasal cavity.

## Results

### Clinical and biochemical manifestations

A total 14 patients were included in our current study with 6 female (42.9%) and 8 male (57.1%). At the time of surgery, the ages of these 14 patients ranged from 29 to 61 years, with a mean age of 46.4 ± 8.9 years. None of the patients had undergone biopsy or craniotomy prior to EEA. In patients who showed visual symptoms, neuro-ophthalmological examination with visual field assessment data was performed. Six patients (42.9%) suffered from preoperative visual deterioration, and all patients were subjected to routine endocrinological testing. In addition, 6 patients (42.9%) had preoperative endocrine abnormalities. Additional clinical characteristics can be found in **Table 1**. The tumor size, based on MRI, ranged from 2.8 to 12.6 cm^3^ with a mean volume of 8.2 ± 3.2 cm^3^. Obstructive hydrocephalus occurred in 3 cases due to space occupation in the third ventricle.

### Tumor removal

**Figure 4:**
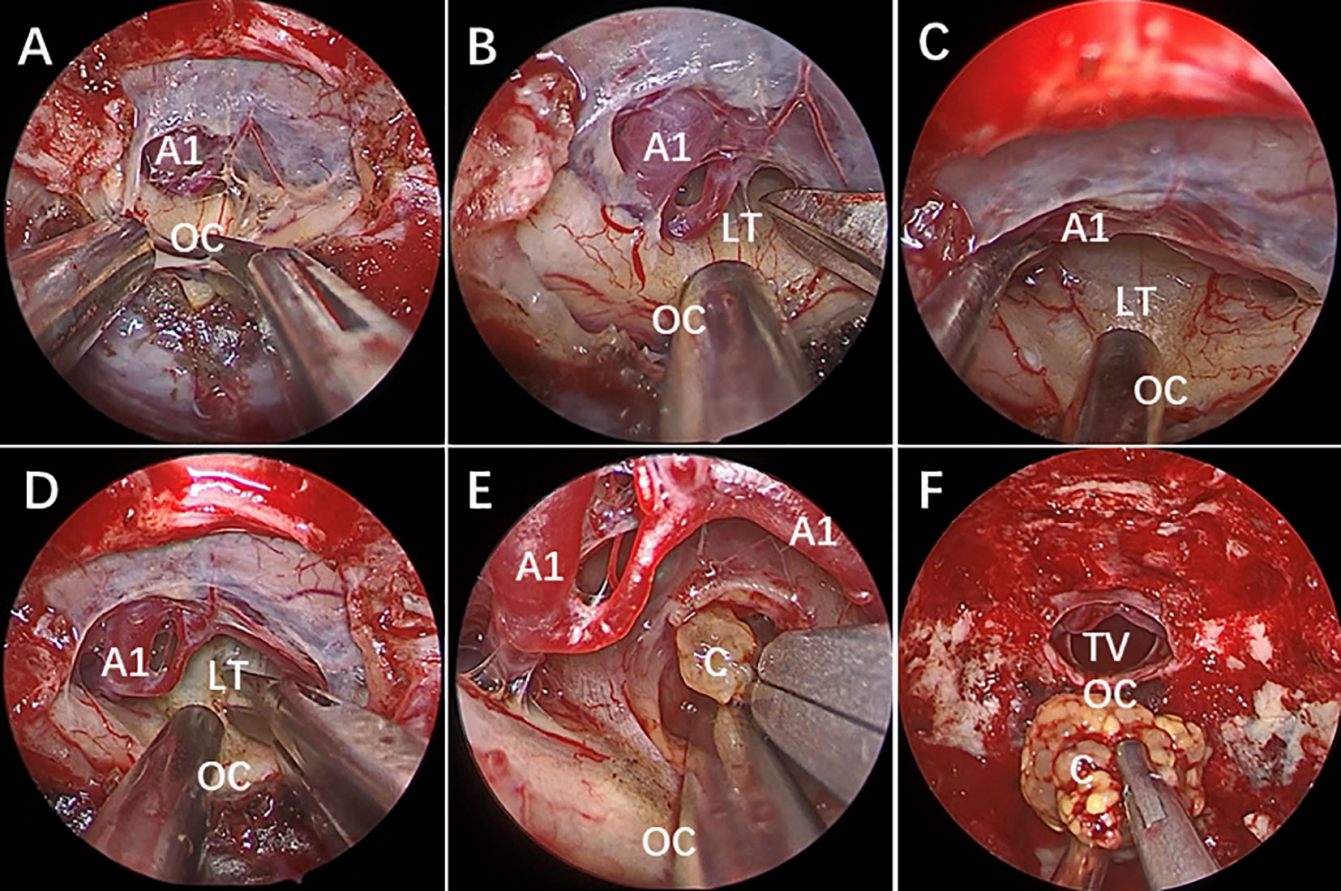
Endoscopic endonasal view of craniopharyngioma resection procedures. **(A)** The optic chiasm exposure after the dura mater opening. **(B)** Lamina terminalis exposure after anterior longitudinal division and anterior circulation artery arachnoid membrane dissection. **(C)** Pulling the optic chiasm downward and the anterior circulation artery system upward to fully exposed the lamina terminalis and the surgical approach, and no tumor was found in the narrow infrachiasmatic space. **(D, E)** The anterior part of the third ventricle tumor was exposed after lamina terminalis incision, and the tumor was debulked and removed using suction tube and grasping forcep piece by piece; **(F)** Complete removal the third ventricle tumor. OC, optic chiasm; A1, A1 segment of anterior cerebral artery; LT, lamina terminalis; TV, third ventricle; C, craniopharyngioma.

**Figure 5:**
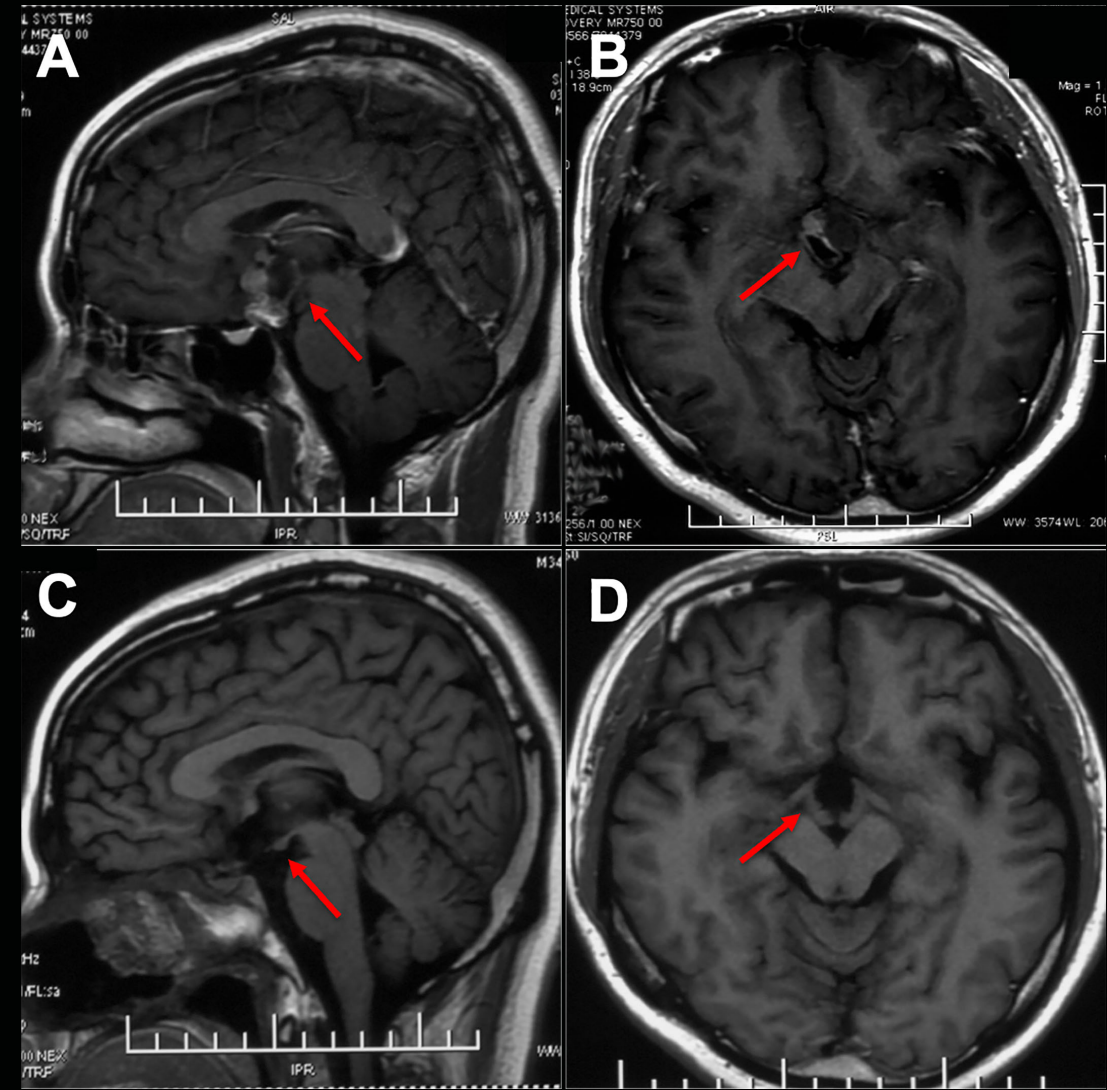
Preoperative and postoperative imaging of ITVC and intact mammary body. **(A, B)** There was a cystic and solid lesion on the suprasellar region. **(C, D)** The optic tract, third ventricle floor and mammillary bodies were well protected postoperatively on MR images.

All the patients were treated with EEEA *via* STLT (**Figure 4**). The postoperative follow-up was conducted at 1 month after surgery, every 3 months during the first year, and every year thereafter. Postoperative MRI showed gross-total resection in 13 of 14 (92.9%) patients (**Figure 5**). Considering the adherence and adjacency of the craniopharyngioma, 1 patient underwent near-total resection of the tumor and gamma knife surgery afterward for the remnant. The tumor had completely disappeared one year later. Obstructive hydrocephalus improvement was observed in 3 patients due to tumor resection and lamina terminalis ostomy. The pathology results showed 9 cases of papillary craniopharyngioma and 5 cases of adamantinomatous subtype. After a mean follow-up of 26.2 months (range 6–54 months), tumor recurrence was not observed (**Figures 1C, D**).

### Endocrine and visual outcome

Postoperatively, 2 patients with preoperative endocrine abnormalities developed panhypopituitarism and received continuous hormone replacement therapy. The other 4 patients showed transient endocrine disorder that lasted for 3 to 6 months and returned to normal, as evidenced by endocrine test retests. The remaining 8 patients with normal endocrine preoperatively did not receive additional hormone replacement therapy after surgery. Transient diabetes insipidus was found in 4 cases, and recovery was achieved with oral desmopressin within 2 months. No new anterior pituitary dysfunction, permanent diabetes insipidus or hypothalamic obesity was found. The variation between pre- and postoperative body mass index (BMI) was less than 4 in all patients (**Table 2**).

**Table 2:** Post-operative clinical characteristics of patients. .

#	Post-op Endocrine deficit (Duration)	Subsequent treatment	Visual change	HC		Nasal exudation	Hospitali-zation	Follow-up
**1**	Panhypopituitarism	Continuous HRT	None		P	3 days	7 days	60 months
**2**	None	None	None		P	2 days	4 days	28 months
**3**	HG (3 months)	Transient HRT	Improved		P	5 days	7 days	36 months
**4**	None	None	None		Ad	4 days	5 days	36 months
**5**	None	None	None		P	2 days	5 days	30 months
**6**	Panhypopituitarism	Continuous HRT	None		P	3 days	7 days	36 months
**7**	HT (3 months) and DI (2 weeks)	Transient HRT and oral desmopressin treatment	None		Ad	4 days	6 days	30 months
**8**	None	None	None		P	3 days	5 days	24 months
**9**	HT (3 months) and DI (6 weeks)	Transient HRT and oral desmopressin treatment	None		Ad	1 days	4 days	24 months
**10**	None	None	Improved		P	4 days	6 days	22 months
**11**	HG (6 months)	Transient HRT	VD		P	4 days	7 days	18 months
**12**	None	None	None		Ad	3 days	5 days	9 months
**13**	None	None	Improved		P	2 days	5 days	8 months
**14**	None	None	None		Ad	5 days	7 days	6 months

HC, histological classification; HT, hypothyroidism; HG, hypogonadism; HRT, hormone replacement therapy; P, Papillary; VD, visual deterioration; Ad, Adamantinomatous.

In 8 patients with normal vision preoperatively, 7 patients had stable vision following tumor resection, and 1 patient showed visual deterioration and regained normal vision after receiving 3 months of neurotrophic and hyperbaric oxygen treatment. There were 6 patients who presented compromised vision preoperatively; 3 of them exhibited significant vision improvement postoperatively, while the other 3 patients did not have any deterioration or improvement postoperatively and were classified as stable. Overall, vision remained stable or improved in 13 (92.9%) patients following surgery. Aside from the visual deterioration referenced above, there were no instances of neurological decline or frontal lobe-related and vascular complications (**Table 2**).

### Efficiency of skull base reconstruction

**Figure 6:**
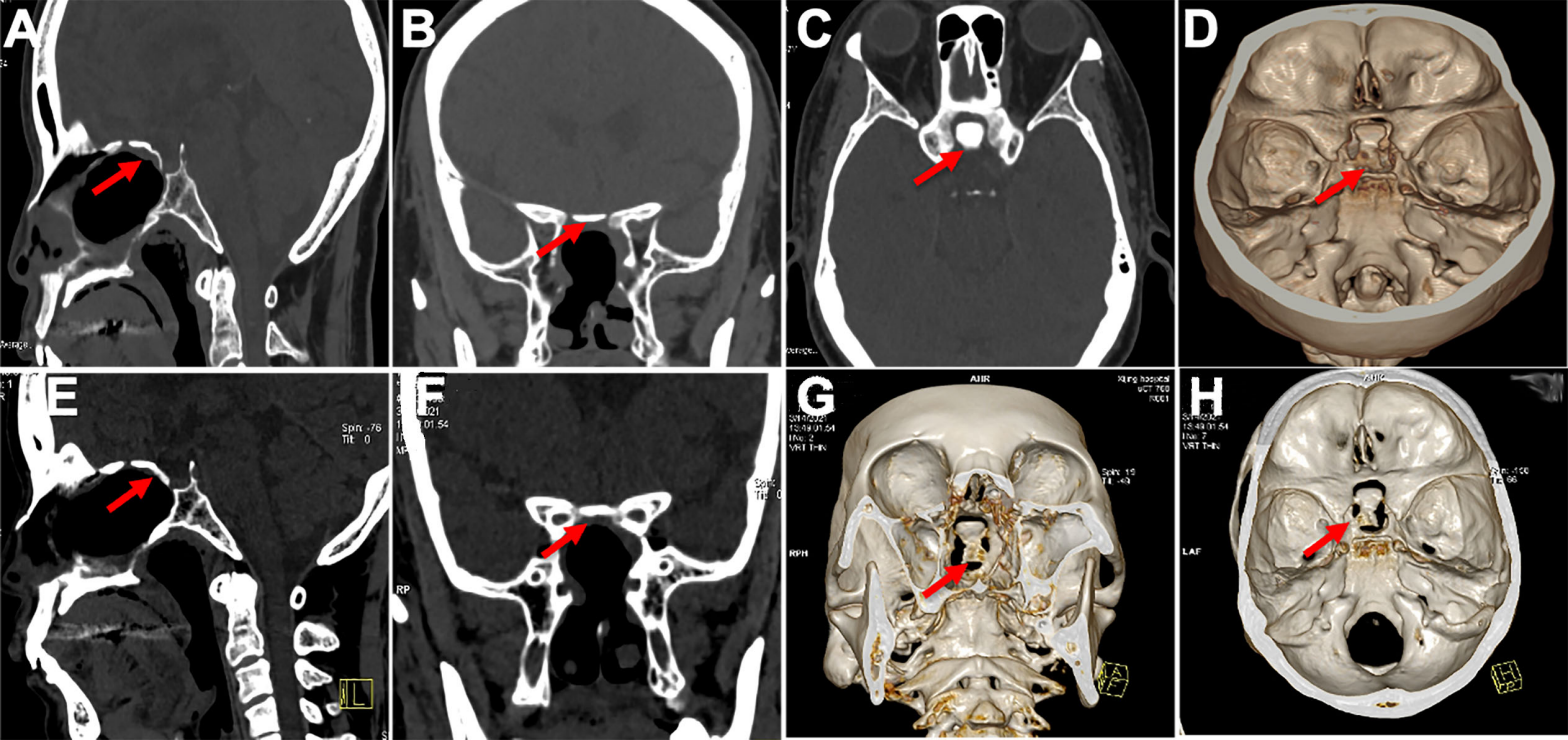
Displayed in situ bone flap postoperatively in 3D reconstruction. **(A–D)** In situ bone flaps were in good position and achieved complete osseous reconstruction 6 months after surgery. **(E–H)** The bone flap was not absorbed, and there was new ossification between the bone flap and surroundings on CT images 18 months after surgery.

**Figure 7:**
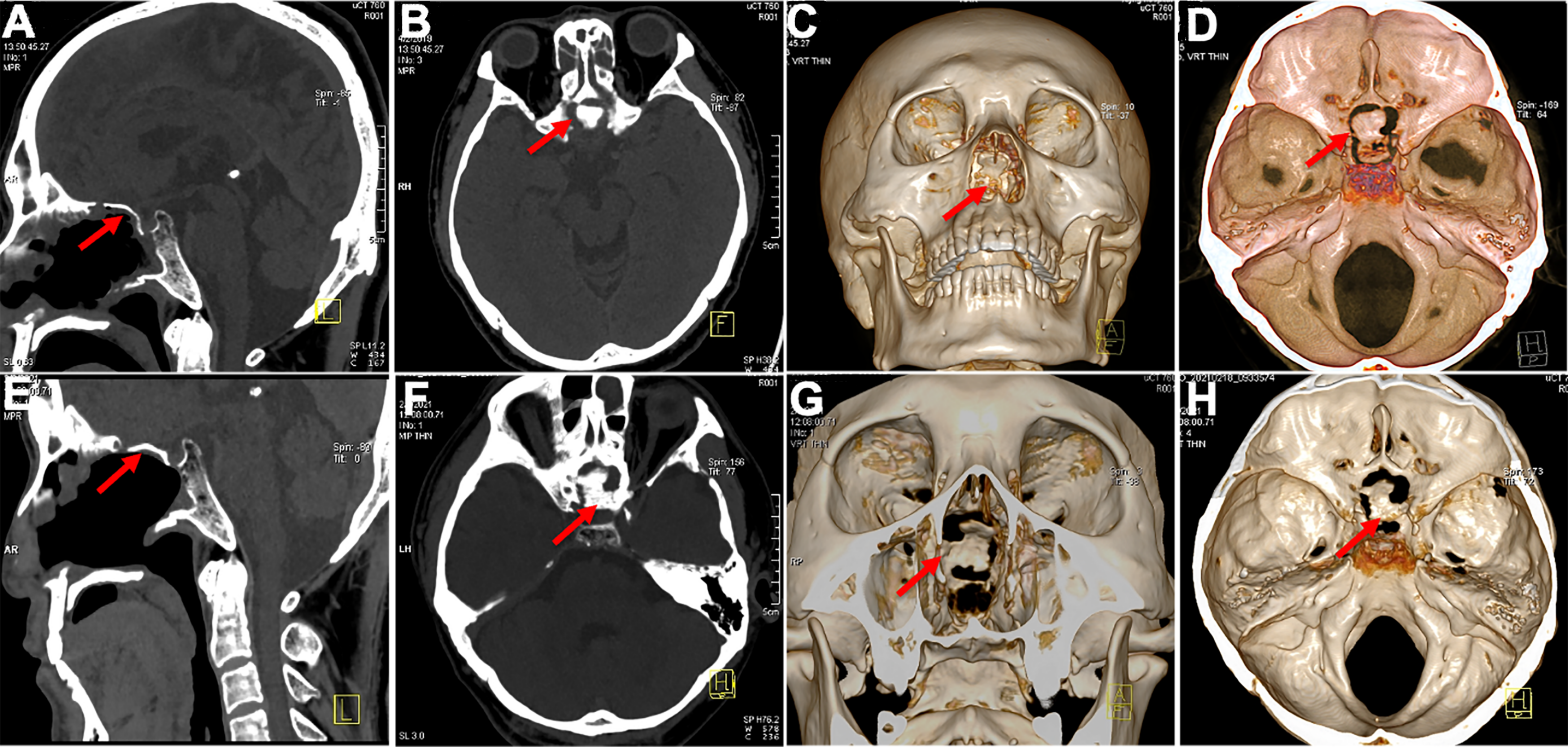
Postoperative bone flap display. **(A, B)** The bone flap in good position 3 months after surgery **(C, D)** achieved complete osseous reconstruction (3 months postoperatively). **(E, F)** CT images showed in situ bone flap 3 years postoperatively and still in the original place. **(G, H)** Obvious and fresh bone formation around the bone flap 3 years postoperatively.

No CSF leakage was observed. Intracranial and pulmonary infections were also not found. The mean durations of nasal exudation and hospitalization were 3.2 days and 5.7 days, respectively. The skull base rebuilt using *in situ* bone flap reconstruction was satisfied, according to 3D reconstruction data. The bone flaps remained in their original position and were not absorbed 18 months postoperatively (**Figure 6**). The bone flap achieved complete osseous reconstruction 3 months after surgery (**Figures 7A–D**) and the formation of large fresh bone was observed between the bone flap and surrounding structures on 3D CT reconstructions 3 years after surgery (**Figures 7E–H**).

## Discussion

The craniopharyngioma can be treated *via* either craniotomy resection or endonasal approach, yet the preference of these two alternatives are still under debate. The ITVC accounts for 0.7%-11% of all craniopharyngiomas (15). Most ITVCs in adults are papillary subtypes and tend to be solid tumors without calcification (16). They commonly originate from the junction of the hypothalamus and stalk and grow upward along the hypothalamus (17). Craniopharyngiomas have been classified based on several criteria, including their relationship to the OC, diaphragma sellae, third ventricle and infundibulum. Steno (18) classified craniopharyngiomas into intraventricular and extraventricular types. Type IV is defined as exclusively located in the third ventricle that does not penetrate the third ventricular floor and is not accessible *via* an endonasal approach, according to Kassam’s classification based on the relative position of the infundibulum (19). Due to its anatomical characteristics, radical removal of craniopharyngioma against tumor recurrence increases the possibility of jeopardizing the topographically adjacent structure hence the function reservation, and appropriate approach used for tumor exposure is the key factor for operational procedures. Although the traditional EEEA creates extended operative working angles for maximizing tumor resection while minimizing surgical blind spots, improving resection degree, visual outcomes and prognosis, and reducing complication incidence and hospitalization duration (20–22), resection *via* endonasal approach can damage vital structures such as the infundibulum and papillary body. Hence, most of the ITVC are treated by craniotomy *via* either interhemispheric translamina terminalis or transcallosal approach (15), which require brain retraction and neurovascular structures irritation along the surgical corridor (23).

Cavallo et al. described the following anatomic conditions that can affect the endonasal approach to the ventricular area: chiasm position, chiasm-pituitary gland distance, the TVF, and distance between the mammillary bodies and dorsum sellae (23). The position of the chiasm is the most important factor in approaching the ventricle *via* the endonasal route. Superior displaced chiasm indicates a wide space between the chiasm and the stalk, which can be a good corridor to the ventricular floor (**Figure 2E, F**). The tumor was exposed directly after opening the dura mater and arachnoid membrane. The superior hypophyseal arteries, passing medially to the pituitary stalk, OC, infundibulum and TVF, should always be preserved to avoid damage to vision and the hypothalamus. The OC descended after infrachiasmatic tumor decompression, making more space in the suprachiasmatic corridor, so that the STLT corridor was available for residual tumor resection.

For the anterior ITVC, the chiasm is displaced inferiorly and the infrachiasmatic space is limited (**Figure 2A**). In our present study, the bulged and elongated LT is the optimal therapeutic approach that points directly towards the upper part of the third ventricle so that larger superior and anterior extension could be achieved for intraventricular tumors (7) (**Figures 2B, C**). The endonasal endoscopic approach *via* LT enabled direct access to the long axis of the tumor, early tumor debulking, better surgical visualization and little damage to the mammillary body, ventricular floor and stalk (**Figure 2D**). The AComA, in our present study, is a uncertainty in LT approach resection as it could narrow the posterior third ventricle exposure, leading to subtotal tumor resection. Visual impairment was reported in both the EEA and transcranial cohort (10), and postoperative visual worsening occurs in approximately 2%–7% of patients (24, 25). The OC blood supply was mainly delivered from the inferior side of OC, and there was no superior supply in the central part, which prevented the STLT corridor from injuring the blood supply (**Figures 3A, B**), making STLT a more favorable approach in protecting the OC than the infrachiasmatic corridor. Meanwhile, in the current study, no extra visual impairment observed in patients with STLT treatment, nor obvious damage to the optic nerve or supply artery during surgery. Also, the occurrence of hypothalamic injury and diabetes insipidus were greatly reduced, indicating better pituitary stalk protection.

Skull base reconstruction is an important assurance of the endoscopic approach application as it could prevent severe post-op complications such as cerebrospinal fluid leaks, tension pneumocephalus and meningitis. The basic premise of reconstruction is to provide enough initial resistance against CSF pressure to allow the grafts to adhere and heal. A variety of reconstructive techniques have been described in the skull base literature, among which the multilayered reconstruction technique has always been a classical repair method (26). Mucosalization is important for skull base defect healing, and after the application of pedicled nasoseptal flap developed by Haddad, there is a striking decrease in CSF leak rates in the endonasal approaches series, and EEEA has become more popular among clinicians (27). Different kinds of skull base reconstructions are tested for craniopharyngioma treatment. The multilayer reconstruction technique (fat, fascia lata inlay and onlay, nasoseptal flap or Dura Seal) is a predominantly applied method in many medical centers (28, 29). A retrospective study suggested that buttresses are beneficial for the repair of most grade CSF leaks. Additionally, the study suggested that it is useful to harvest septal bone or vomers if available during the nasal phase (26). The skull base is uneven at the planum sphenoidal joint of the anterior skull base and sellar floor. In any case, the tailored bone cannot fit the skull base deficit perfectly, which may be attributed to the cause of CSF leakage and neurovascular structure damage, as it may slide into the cranial cavity.

In the current study, we recommended four levels of skull base repair protocol: for the first layer (**Figure 3D**), place an artificial biomembrane lining the inner surface of the dura and completely cover the defect area. For the second layer (**Figure 3E**), an *in situ* bone flap is placed over the dura mater to achieve osseous reconstruction. We innovatively used grinding drills to make *in situ* bone flaps (tuberculum-planum-seller micro bone flaps) for deficit repair. An *in situ* bone flap is easy to harvest, can fit well with the skull base, and can maximally restore anatomical structure with little risk of histological rejection. The application of *in situ* bone flap repair could efficiently convert high-flow CSF leaks into low-flow CSF leaks, which is essential for watertight closures and in general accelerates the recovery of patients. In addition, the extent of dura mater was resected smaller than that of the bone defect, which prevented the bone flap from sliding into the intracranial space. The pedicled nasoseptal flap using mucosal flap with or without fascia lata covered the bone flap (**Figure 3F**). Finally, supporting materials such as gelatin sponge or silver gauze are used to pack the nasal cavity. Patients in our current study showed satisfied reconstruction results without CSF leakage, indicating a novel and promising method for skull base reconstruction after endoscopic surgery.

There are a few limitations in this study. 1). we mainly analyzed the anterior ITVC, a rare subtype, in a retrospective study, and the small number of clinical cases makes it difficult to draw well-founded conclusions. Further prospective, randomized, multi-institutional collaboration and larger volume series are needed to develop and refine the surgical nuances and reconstruction strategies to fully evaluate this approach for ITVC. 2). suprasellar craniopharyngiomas that break through the floor of the third ventricle superiorly were not discussed. 3). there is great heterogeneity in the operative care of craniopharyngiomas across different institutions (30), with limited evidence regarding comparative complications. The low incidence of postoperative complications may be partially attributed to the relatively smaller tumor volume in the data.

## Conclusion

In conclusion, patients with anterior ITVC can be treated effectively and safely *via* STLT under EEEA with satisfied resection rate as well as low incidence of pituitary and visual complications. The clinical efficacy of the *in situ* bone flap reconstruction technique is an effective method in achieving watertight closure after EEEA and can decrease meningitis, hospitalization and extra revisits to the operating room.

## Data availability statement

The raw data supporting the conclusions of this article will be made available by the authors, without undue reservation.

## Ethics statement

This study was reviewed and approved by Ethics Committee of Fourth Military Medical University. The patients/participants provided their written informed consent to participate in this study. Written informed consent was obtained from the individual(s) for the publication of any potentially identifiable images or data included in this article.

## Author contributions

YZ and DG conceived the design of this study. YZ and JW drafted the manuscript and modified the details. JW, YH, TJ, JL, PJ and SY collected patient data and performed the analysis. XJ and WL trimmed the design of the study. All authors contributed to the article and approved the submitted version.

## Funding

This research was supported by the Natural Science Foundation of China (NO. 81971227).

## Conflict of interest

The authors declare that the research was conducted in the absence of any commercial or financial relationships that could be construed as a potential conflict of interest.

## Publisher’s note

All claims expressed in this article are solely those of the authors and do not necessarily represent those of their affiliated organizations, or those of the publisher, the editors and the reviewers. Any product that may be evaluated in this article, or claim that may be made by its manufacturer, is not guaranteed or endorsed by the publisher.

## Supplementary material

A video illustrating the surgical steps and technical nuances of skull base reconstruction with an *in situ* bone flap for the ITVC was uploaded to YouTube on February 13, 2018. The original bone flap reconstructs the skull base after endoscopic endonasal resection of craniopharyngioma in the third ventricle (Dakuan Gao, Xijing Hospital, China) (https://youtu.be/rZKq_EpDRMA).
